# Global analysis of gene expression mediated by OX_1_ orexin receptor signaling in a hypothalamic cell line

**DOI:** 10.1371/journal.pone.0188082

**Published:** 2017-11-16

**Authors:** Eric Koesema, Thomas Kodadek

**Affiliations:** Department of Chemistry, The Scripps Research Institute, Scripps Florida, Jupiter, FL, United States of America; Universidad de la Laguna, SPAIN

## Abstract

The orexins and their cognate G-protein coupled receptors have been widely studied due to their associations with various behaviors and cellular processes. However, the detailed downstream signaling cascades that mediate these effects are not completely understood. We report the generation of a neuronal model cell line that stably expresses the OX_1_ orexin receptor (OX_1_) and an RNA-Seq analysis of changes in gene expression seen upon receptor activation. Upon treatment with orexin, several families of related transcription factors are transcriptionally regulated, including the early growth response genes (*Egr*), the Kruppel-like factors (*Klf*), and the *Nr4a* subgroup of nuclear hormone receptors. Furthermore, some of the transcriptional effects observed have also been seen in data from *in vivo* sleep deprivation microarray studies, supporting the physiological relevance of the data set. Additionally, inhibition of one of the most highly regulated genes, serum and glucocorticoid-regulated kinase 1 (*Sgk1*), resulted in the diminished orexin-dependent induction of a subset of genes. These results provide new insight into the molecular signaling events that occur during OX_1_ signaling and support a role for orexin signaling in the stimulation of wakefulness during sleep deprivation studies.

## Introduction

The orexin system has been shown to influence several biological processes including appetite [1–3], wakefulness [4–10], reward behaviors [11–18], and energy metabolism [19–24]. This system consists of two G-protein coupled receptors (GPCRs), the OX_1_ orexin receptor (OX_1_) and the OX_2_ orexin receptor (OX_2_), and a pair of hypothalamic peptide agonists, orexin A (OxA) and orexin B (OxB) [1]. The broad range of biological effects of the orexin system are attributed to widespread projections of orexigenic neurons and broad expression patterns of the receptors throughout the central nervous system [25].

In addition to efforts demonstrating the behavioral effects of the orexin system, a number of studies have addressed the intracellular molecular signaling events that occur in response to orexin receptor activation [26–30]. Upon ligand binding, these receptors can couple to various G-proteins [31–33] and regulate diverse signaling mechanisms including calcium influx [34–37], adenylyl cyclase/cAMP [38,39], PI3K [40], MAPK/ERK [30,41,42], and several phospholipases [43], as recently reviewed [44–46]. While these signaling studies have provided much insight, they are largely focused on the upstream, canonical GPCR signaling pathways, leaving the detailed downstream signaling cascades unclear. Furthermore, most of these signaling studies have been done in heterologous expression systems that are non-neuronal, resulting in varied responses and raising questions of applicability to orexin receptor signaling in a neural context [37,47–55].

In this study, we report the generation a of a neuronal cell line that stably expresses OX_1_ and the transcriptional profile seen upon receptor activation, as determined by RNA-Seq. OX_1_ activation resulted in the differential regulation of a large set of genes, several of which have previously been shown to be similarly regulated by sleep deprivation (SD), *in vivo*. Additionally, the downstream role of one of the more highly regulated genes, *Sgk1*, was further characterized.

## Materials and methods

### Cell culture

The following cell lines were acquired from ATCC: CHO-K1 (CRL-9618), Neuro-2a (CCL-131), SH-SY5Y (CRL-2266), and AR42J (CRL-1492). GT1-7 cells were a kind gift from the lab of Pamela Mellon (University of California, San Diego), while CHO cells stably expressing OX_1_ were generously provided by the lab of Patricia McDonald (The Scripps Research Institute). All cells were grown in the presence of 10% HI-FBS (Gibco) at 37°C, 5.0% CO_2_. Base media for each cell line are as follows: DMEM, high glucose (Gibco) for GT1-7 and CHO cells, Eagle's Minimum Essential Medium (ATCC) for Neuro-2a (N2A) and SH-SY5Y cells, and F-12K Medium (ATCC) for AR42J cells.

### Analysis of mRNA transcripts by qPCR

Cells were grown to near confluence in 60mm culture dishes. For orexin treatments, culture media was replaced with fresh, warm media containing 100nM orexin A (OxA, Tocris) and incubated for 3h at 37°C. For OX_1_ inhibition, cells were pretreated with media containing 3μM SB-334867 (Tocris) for 10 min prior to adding OxA. RNA was purified from cells with the RNeasy Plus Mini Kit (Qiagen). First strand cDNAs were synthesized with the iScript cDNA Synthesis Kit (BioRad) in a 20μl reaction using 1μg RNA. The qPCR reactions were done in triplicate on the StepOnePlus real time PCR system (Applied Biosystems) with TaqMan gene expression assays (Applied Biosystems) using the following conditions: 1μl cDNA, 1μl TaqMan probe (S1 Table), 10ul TaqMan Gene Expression Master Mix (Applied Biosystems), and 8ul nuclease-free water. Thermal cycling conditions were 95°C for 10m, then 40 cycles of 95°C for 15s and 60°C for 60s.

### Inositol phosphate assay

To demonstrate the presence of functional orexin receptors, the IP-One HTRF assay (CisBio) was used with a modified protocol. Cells were harvested with TrypLE Express (Life Technologies), washed once with Dulbecco's phosphate-buffered saline (Gibco, 2.67mM KCl, 1.47mM KH_2_PO_4_, 137.93mM NaCl, 8.06mM Na_2_HPO_4_-7H_2_O), resuspended in 1X Stimulation Buffer (CisBio, 10mM Hepes, 1mM CaCl_2_, 0.5mM MgCl_2_, 4.2mM KCl, 146mM NaCl, 5.5mM glucose, 50mM LiCl, pH 7.4), and plated in 7μl aliquots into a low-volume 384-well plate (white) at a concentration of 20,000 cells per well. Serial dilutions of the orexin-A peptide (OxA, Tocris) were made at 2x final concentration in 1X Stimulation Buffer and then added to the cells in a 1:1 ratio (7μl per well). After 45 minutes at 37°C, the HTRF reagents were added (3μl each) and the plate was incubated for 1h at room temperature.

For the OX_1_ inhibition assay, 4-fold serial dilutions of SB-334867 were done in DMSO, starting from 2.5mM. 7μl of each serial dilution was then added to 293μl 1X Stimulation Buffer. These dilutions were then added in a 1:1 ratio (v/v) to each well of a low-volume 384-well plate (white) already containing 20,000 GT1-7-OX_1_ cells per well in 6μl 1X Stimulation Buffer. After a 30 minute incubation at 37°C, 2μl OxA (varying concentrations in 1X Stimulation Buffer) was added to each well (final [DMSO] = 1%). After 45 minutes at 37°C, the HTRF reagents were added (3μl each) and the plate was incubated for 1 hour at room temperature. Data were acquired on a Tecan Infinite M1000 Pro plate reader. All experiments were done in triplicate.

### Generation of GT1-7-OX_1_ stable cells

The gene for human OX_1_ (Genecopoeia, EX-U0062-M02) was subcloned into pCDH-CMV-MCS-EF1-copGFP (Systems Biosciences) at the XbaI and BamHI restriction sites. Lentiviral particles were generated in HEK293T cells (ATCC) by co-transfecting the pCDH-OX_1_ plasmid with the pPACKH1 HIV Lentivector Packaging Kit (Systems Biosciences) using FUGENE HD Transfection Reagent (Promega). The lentiviral particles were then concentrated with PEG-it Virus Precipitation Solution (Systems Biosciences), resuspended in 400μl DPBS + 25mM HEPES, and stored at -80°C until ready for use. For the viral transduction, GT1-7 cells were cultured in 6-well plates to 60% confluence. Various volumes of lentivirus (50, 100, 200μl) were added directly to the culture media and mixed. After 72h, reporter gene expression and cell viability were analyzed via microscopy and the cells that demonstrated the highest levels of GFP expression with minimal cell toxicity were expanded for further analysis.

### RNA-Seq library construction and sequencing

GT1-7-OX_1_ cells were plated in 75cm^2^ culture flasks and grown to near confluence. For cell treatments, culture media was replaced with fresh, warm media containing vehicle (H_2_O) for 8 hours, or 200nM OxA for 3 or 8 hours. RNA was isolated with TRIzol Reagent (Life Technologies) according to manufacturer’s protocol, including the addition of 10μg RNase-free glycogen (ThermoFisher). The RNA samples were then treated with DNase (New England Biolabs) to remove any genomic DNA contamination and then cleaned up with the Purelink RNA Micro Kit (Invitrogen). This process was repeated twice for n = 3 per condition. The DNase-treated Total RNA (250ng) was depleted of ribosomal RNA using the TruSeq Stranded Total RNA kit (Illumina) and quality assessed on an Agilent 2100 Bioanalyzer to confirm that 18S and 28S rRNA peaks were depleted. The rRNA-depleted RNA was converted to dsDNA libraries by following the TruSeq Stranded Total RNA sample prep kit user guide. Briefly, the RNA was fragmented, converted to cDNA, and ligated with adaptors. The adaptor-ligated DNA was then PCR amplified using 11 cycles to generate the final libraries. The final libraries were size selected and purified using 1.0 x Ampure XP beads (Beckman Coulter) then validated by the Bioanalyzer and qPCR quantified using primers that recognize the Illumina adaptors. The libraries were then pooled at equimolar ratios, quantified using qPCR (quantification of only the adaptor-ligated libraries) and loaded onto the NextSeq 500 flow cell (Illumina) at 1.8pM final concentration. Demultiplexed and quality filtered raw reads (fastq) generated from the NextSeq 500 were trimmed (adaptor sequences) using Trimmomatic, version 0.35 [56] and aligned to the reference genome (UCSC-mm10) using STAR, version 2.5.2a [57]. HTSeq-count (version 0.6.0) was used to generate gene counts and differential gene expression analysis was performed using DESeq2 (version 1.10.1, R version: 3.2.3) [58], comparing the OxA-treated samples to those treated with vehicle. The principle component analysis was performed via the plotPCA function in DESeq2, using the regularized log-transformed values of the 500 genes that were the most variable across all samples. In order to more closely identify the relationship between each sample and every other sample, the Euclidean distance between each pair of samples was calculated using the log-transformed values of the complete data set. Complete linkage clustering was then used to generate a sample-to-sample distance heatmap, via the pheatmap package in R source. For statistical analyses, raw counts for the two conditions of interest were imported into DESeq2 and transformed using the negative binomial Wald test. Adjusted p-values were generated via the Benjamini-Hochberg procedure.

### Promoter analysis

Gene symbols of the differentially expressed genes identified in the RNA-Seq data were entered into the DAVID Gene ID Conversion tool (https://david.ncifcrf.gov/) [59,60] under the settings “OFFICIAL_GENE_SYMBOL” (input) and “REFSEQ_MRNA” (output). From the complete set of 332 genes that were differentially regulated 2-fold or greater by OX_1_ signaling at 3 or 8h (adj. p-values <0.05, log_2_ Fold Change (log_2_FC) >1.0 or < -1.0), there were 31 official gene symbols that either were not recognized by DAVID, or could not be converted into a RefSeq_mRNA ID recognized by PSCAN, and could not be included in the PSCAN analysis. The complete list of gene ID conversions, including the unrecognized gene ID’s, can be found in S2 Table. The remaining gene ID’s were entered into the PSCAN user interface (http://159.149.160.88/pscan/), and run with the following settings: Mus musculus (organism), -450 +50 (region), Jaspar 2016 (Descriptors).

### Comparison between GT1-7-OX_1_ RNA-Seq and SD microarray meta-analysis

The RNA-Seq data from this work were cross-compared to results from an SD microarray meta-analysis [61]. Of the 91 SD-related mouse genes presented in the Wang, et al. study, five did not have Gene ID’s that correlated to our data set (*2310076G05Rik*, *3110003A17Rik*, *4932442K08Rik*, *C330006P03Rik*, and *D930028F11Rik*) and were not included in the comparison. Notably, our data set does not distinguish between the long and short isoforms of *Rbm3*, which were oppositely regulated by SD in the meta-analysis. Therefore, we also excluded *Rbm3* for this comparison.

### Sgk1 inhibition assay

GT1-7-OX_1_ cells were plated in 75cm^2^ culture flasks and grown to 70–90% confluence. Growth media was replaced with fresh, warm media containing 1.0μM GSK-650394 (Apexbio Technology), or DMSO vehicle. After 30 minutes at 37°C, either H_2_O vehicle or OxA was added to the media at 200nM (final). After another 3 hours of incubation at 37°C, RNA was purified from cells with the RNeasy Plus Mini Kit (Qiagen). First strand cDNAs were synthesized with the iScript cDNA Synthesis Kit (BioRad) in 400μl reactions using 20μg RNA. The qPCR reactions were done with PowerUp SYBR Green Master Mix (Applied Biosystems) and PrimeTime qPCR primer pairs (IDT, S3 Table) in 20μl reactions (10μl SYBR, 2μl primer pair (500nM, final), 0.5μl cDNA), in triplicate, on the StepOnePlus real time PCR system (Applied Biosystems). Cycling conditions were 50°C for 2 minutes, 95°C for 2 minutes, then 40 cycles of 95°C for 15 seconds, 50°C for 1 minute. In addition to 89 genes of interest, primer pairs targeting seven housekeeping genes were included (*Actb*, *B2m*, *Gusb*, *Polr2a*, *Ppia*, *Rplp0*, and *Tbp*). As it demonstrated the strongest stability amongst treatments, with a geNorm M value <0.2 (as determined via qbase+ software, version 3.1), *B2m* was used as the endogenous control for data analysis. Data were analyzed by the 2^-ΔΔC^_T_ method and represented as fold-change over control samples.

## Results

### Characterization of orexin receptor-expressing cell lines

**Fig 1:**
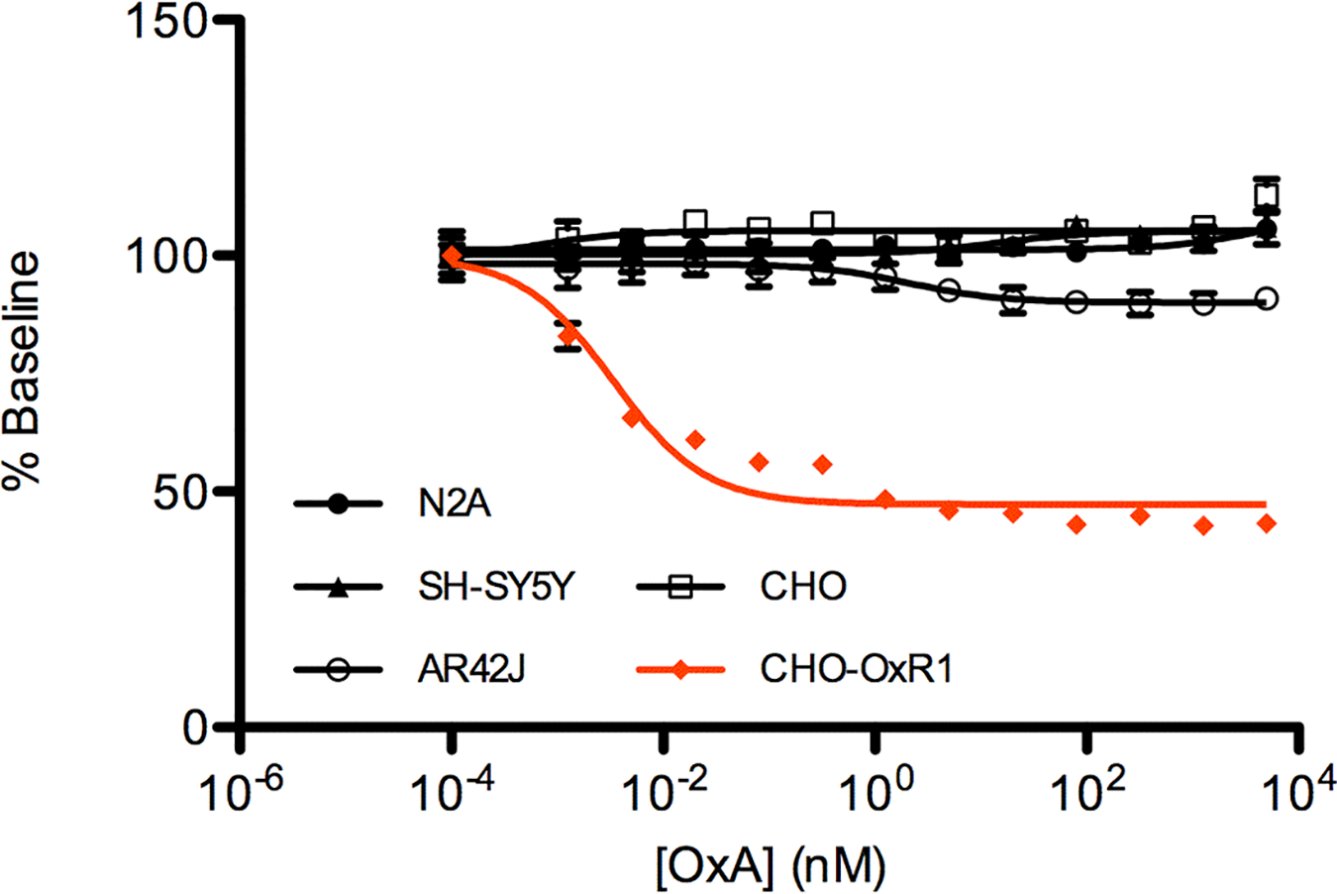
Characterization of cell lines previously reported to express orexin receptors. Each cell line was assayed for the presence of functional orexin receptors via the IP-One HTRF Assay. Cells were incubated with orexin A at various concentrations for 45 min. A CHO-based cell line stably expressing OX_1_ (CHO-OX_1_) was used as a positive control. The data are presented as a percentage of the baseline HTRF ratio (A_665_/A_620_ x 10000). Data points are mean (n = 3) and error bars represent standard error of the mean (SEM).

The initial goal of this study was to identify a cell line that would be a reasonable model in which to analyze OX_1_ signaling. To that end, several cell lines, originating from different species, that have been reported to express one or both of the orexin receptors, endogenously, were acquired [51,62–64]. Each cell line was screened for the presence of OX_1_ and OX_2_ mRNA by qPCR with a set of probes designed to span various exons (Table 1). While some amplification was observed sporadically, the high Ct values and inconsistency between probes did not clearly demonstrate the presence of orexin receptor transcripts in any of the cell lines tested. In order to look for the presence of functional orexin receptors, the IP-One HTRF assay was employed. This assay is a FRET-based immunoassay that measures accumulation of inositol monophosphate (IP1) upon activation of the phospholipase C pathway and is often used as a measure of G_q_-coupled GPCR activation. At baseline levels, the kit components (FRET donor and acceptor) are bound together, resulting in a high HTRF ratio. Upon ligand-induced GPCR activation, production of cellular IP1 is stimulated. This native, unlabeled IP1 then displaces acceptor-labeled IP1, disrupting the proximity of the donor and acceptor molecules and resulting in decreased HTRF ratios. While this assay is commonly used to quantify IP1 production (via a standard curve), we utilized it as a simple measure of receptor functionality and did not perform this quantitation. As shown in Fig 1, none of the cell lines tested demonstrated detectable GPCR activation in response to increasing concentrations of the OxA peptide, whereas a CHO-based cell line stably expressing OX_1_ (CHO-OX_1_) provided a robust response.

**Table 1:** Characterization of orexin receptor expression via qPCR.

** **	**Cell Line **	**CHO**	**CHO-OX**_**1**_	**AR42J**
** **	**Species**	Chinese Hamster	Chinese Hamster	rat
** **	**Type**	Ovary	Ovary	pancreatic cancer
** **	**Housekeeping Gene**	Human TBP	Human TBP	Euk. 18S rRNA
** **	**Housekeeping Gene Ct**	27.2247+/-0.0546	27.4827+/-0.0055	14.8803+/-0.0979
**OX**_**1**_	**Probe 1 Ct**	36.5295+/-0.7601	19.4577+/-0.0338	38.8842+/-1.9327
	**Probe 2 Ct**	40.0000+/-0.0000	36.6148+/-0.4199	40.0000+/-0.0000
**OX**_**2**_	**Probe 1 Ct**	36.3154+/-0.106	40.0000+/-0.0000	24.6203+/-0.0351
	**Probe 2 Ct**	32.0488+/-0.0467	32.0777+/-0.036	33.1794+/-0.1724
** **	**Cell Line **	**N2A**	**SHSY5Y**	**GT1-7**
** **	**Species**	mouse	human	mouse
** **	**Type**	neuroblast	neuroblastoma	hypothalamic neuron
** **	**Housekeeping Gene**	Mouse GAPDH	Human TBP	Mouse GAPDH
** **	**Housekeeping Gene Ct**	20.9608+/-0.0221	26.4232+/-0.0538	26.9245+/-0.0194
**OX**_**1**_	**Probe 1 Ct**	27.2944+/-0.0469	33.9322+/-0.3944	35.4889+/-0.1255
	**Probe 2 Ct**	30.5317+/-0.0467	20.8086+/-0.0078	36.6909+/-0.343
**OX**_**2**_	**Probe 1 Ct**	40.0000+/-0.0000	40.0000+/-0.0000	40.0000+/-0.0000
	**Probe 2 Ct**	40.0000+/-0.0000	31.997+/-0.0948	40.0000+/-0.0000

Three cell lines (AR42J, SH-SY5Y, and GT1-7) that have been previously reported to express orexin receptors, plus two negative control cell lines (CHO, N2A) and one positive control cell line (CHO-OX_1_), were screened for the presence of OX_1_ and OX_2_ mRNA via qPCR. Average Ct values are shown +/- standard deviation (n = 1, reads done in triplicate). Undetermined Ct values were assigned a value of 40, the total number of cycles used. The probes used for each sample are listed in S1 Table.

### Generation of GT1-7 cells stably expressing OX_1_

**Fig 2:**
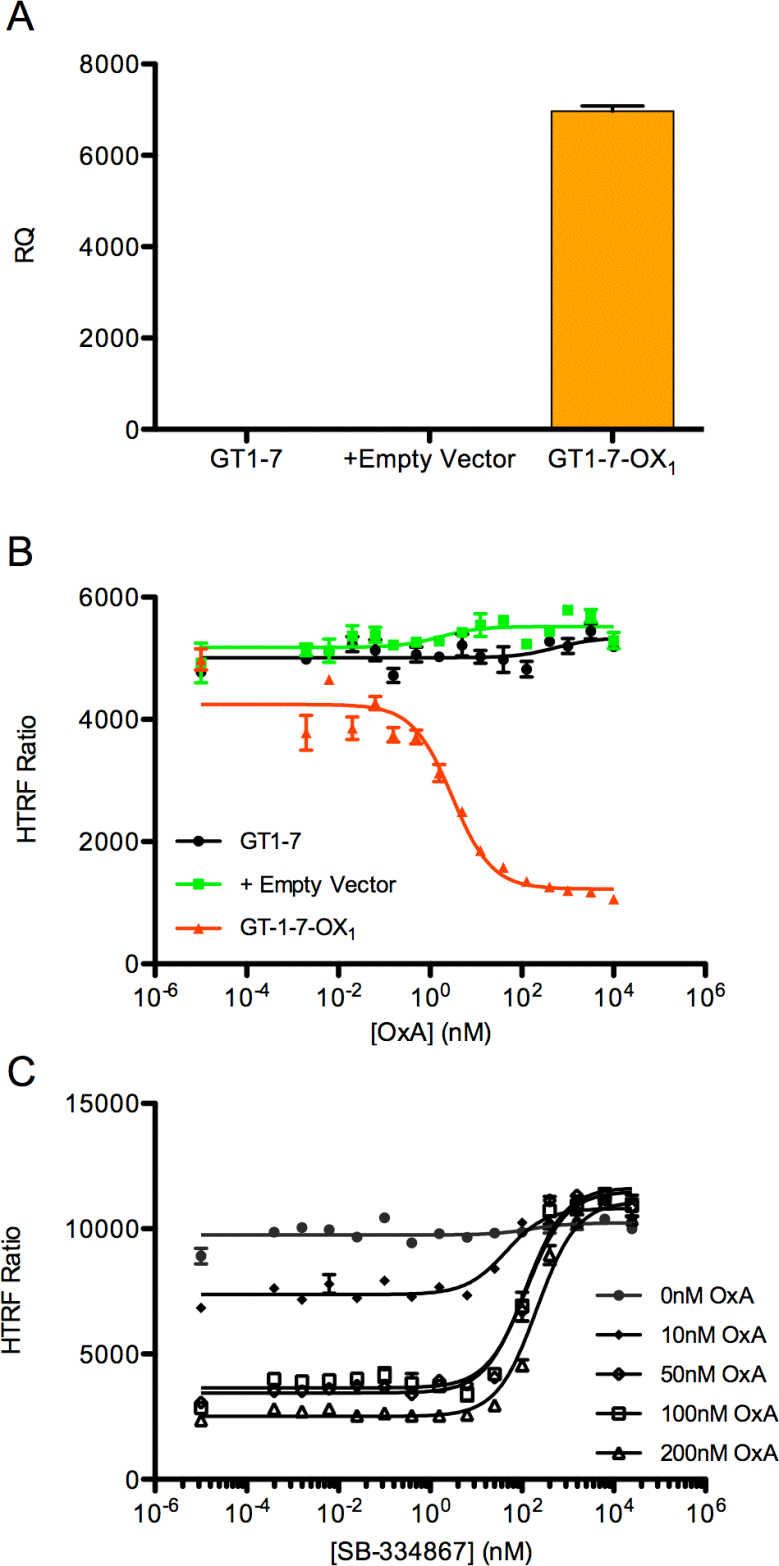
Generation of a GT1-7-based cell line stably expressing OX_1_. A lentiviral transduction system was used to generate GT1-7 cells that stably express OX_1_. (A) Presence of OX_1_ mRNA in the transduced cells was verified via qPCR. Data were analyzed by the 2^-ΔΔC^_T_ method, using mouse GAPDH as the reference, and are expressed as relative quantity (RQ), normalized to the parental cell line. (B) Parental, mock-transduced, and OX_1_-transduced GT1-7 cells were tested for the presence of functional OX_1_ via the IP-One HTRF Assay. (C) An orexin receptor antagonist, SB-334867, blocked orexin signaling in GT1-7-OX_1_ cells in a concentration-dependent manner. Data points are mean (n = 3), error bars represent SEM.

In the absence of an existing cell line that expresses OX_1_ endogenously, we turned to the construction of a neuronal cell line that would express recombinant OX_1_. As the orexin receptors are known to be highly expressed in the hypothalamus [65,66] and GT1-7 is a mouse cell line derived from hypothalamic neurons, it seemed reasonable to use this as the parental cell line. A lentiviral transduction system was used to stably incorporate human OX_1_ into GT1-7 cells. The presence of the transcript was verified by qPCR (Fig 2A) and the presence of functional receptor was demonstrated via IP-One HTRF assay (Fig 2B). The functional response to OxA in these cells was inhibited with SB-334867 [67], a selective inhibitor of OX_1_(Fig 2C).

### RNA Seq analysis

In order to assess changes in gene expression brought about by OX_1_ signaling, GT1-7-OX_1_ cells were treated with OxA for 3 or 8 hours. RNA-Seq analysis was then used to identify transcripts regulated differentially compared to cells treated with vehicle. To evaluate the overall relationships between samples and test for batch effects, a principal component analysis was conducted (Fig A in S1 Fig). In addition, for a more complete analysis of how each sample compares to every other sample, a clustering analysis was performed (Fig B in S1 Fig). In each case, strong clustering among replicates and treatment groups was evident. Additionally, more than 3 x 10^7^ mapped reads were obtained per sample, representing approximately 90% of total reads, while only 0.4% of reads mapped to a ribosomal RNA reference, indicating minimal rRNA contamination. The data discussed in this publication have been deposited in NCBI's Gene Expression Omnibus [68] and are accessible through GEO Series accession number GSE99690 (https://www.ncbi.nlm.nih.gov/geo/query/acc.cgi?acc=GSE99690).

As an initial characterization of the recombinant model, the basal expression of known neural marker genes was analyzed. Using the average transcripts per million (TPM) values of the vehicle-treated control samples as a measure of expression level (Avg. TPM >2.0), GT1-7-OX_1_ cells expressed a number of neuronal marker genes [69], but not glial marker genes, indicating a neuronal genotype (Table 2). Of note, as GT1-7 cells were isolated from mouse hypothalamic tumor cells designed to express the SV-40 T-antigen under the control of the gonadotropin releasing hormone (GnRH) promoter sequence [70], we expected to see elevated levels of GnRH in these cells. As expected, these cells express GnRH at very high levels (Avg. TPM = 3924).

**Table 2:** Expression of neural marker genes in GT1-7-OX_1_ cells.

Neuronal Maker Genes	Average TPM	Glial Maker Genes	Average TPM
Clstn2	115.0	cd68	1.9
TH	69.0	s100b	1.7
ENO2	58.0	pecam1	0.3
DLG4	36.8	cldn5	0.1
Asph	27.0	GFAP	0.1
Vgf	26.1	vwf	0.0
MAP2	21.0	tnf	0.0
Napb	10.5	ocln	0.0
Icam5	10.2	ptprc	0.0
Ttc9	9.8		
Ica1l	8.9		
Pgm2l1	8.8		
Satb2	7.8		
Cxadr	7.1		
Lpl	5.2		
Cacna1b	4.1		
Camk2b	3.7		
SYP	3.3		
Pcsk2	3.1		

To quantify the basal expression levels of known neural marker genes in GT1-7-OX_1_ cells, the TPM values of the vehicle-treated samples were used. Genes were considered to be expressed if the average TPM values were 2.0 or greater.

**Fig 3:**
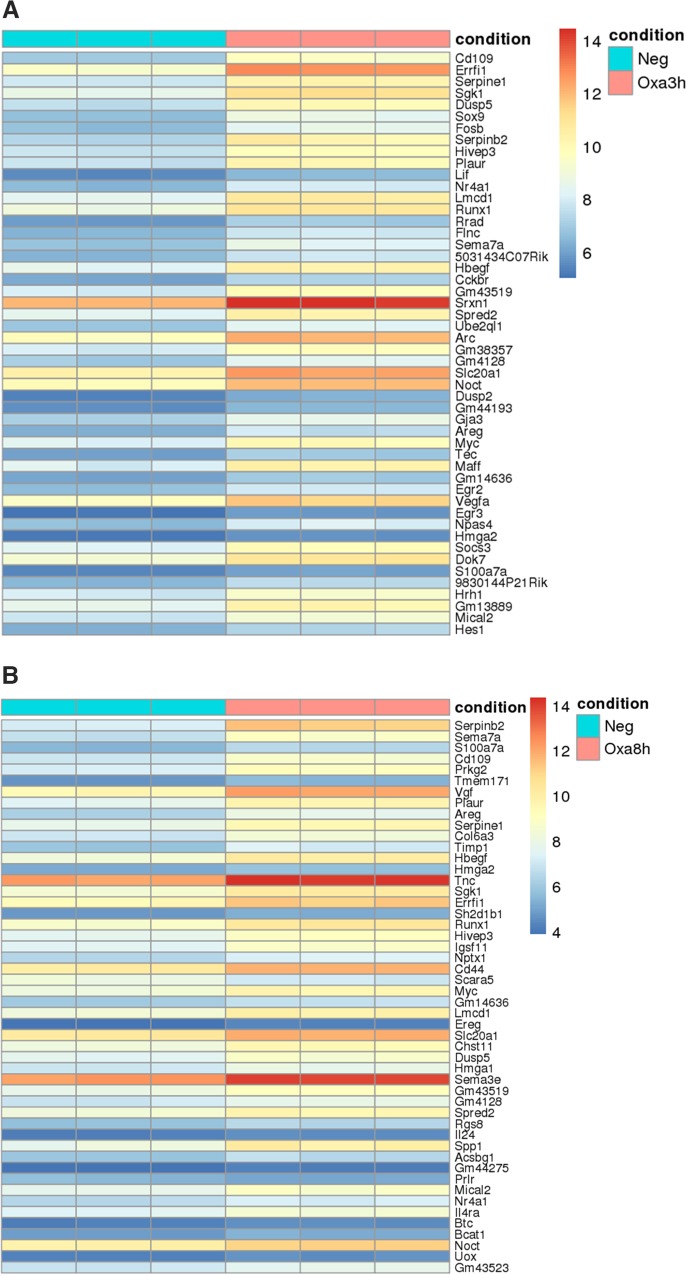
Heat maps indicating the genes most highly regulated by OX_1_ activation. (A) Vehicle-treated vs. 3-hour treatment with OxA. (B) Vehicle-treated vs. 8-hour treatment with OxA. The values (colors) shown are the regularized log transformations of the original count data.

When the GT1-7-OX_1_ cells were treated with the OxA peptide, 5118 genes were differentially regulated with statistical significance at the 3-hour time point (adj. p-values <0.05). Of these, 294 were regulated 2-fold or greater (257 up, 37 down, log_2_FC >1.0 or < -1.0). From the 8-hour OxA treatment, 3683 genes were differentially regulated with statistical significance (adj. p-values <0.05). Of these, 116 were regulated 2-fold or greater (103 up, 13 down, log_2_FC >1.0 or < -1.0). Heat maps were generated to indicate the 50 most differentially regulated genes at each time point (Fig 3), as determined by overall fold change (adj. p-values <0.05).

**Fig 4:**
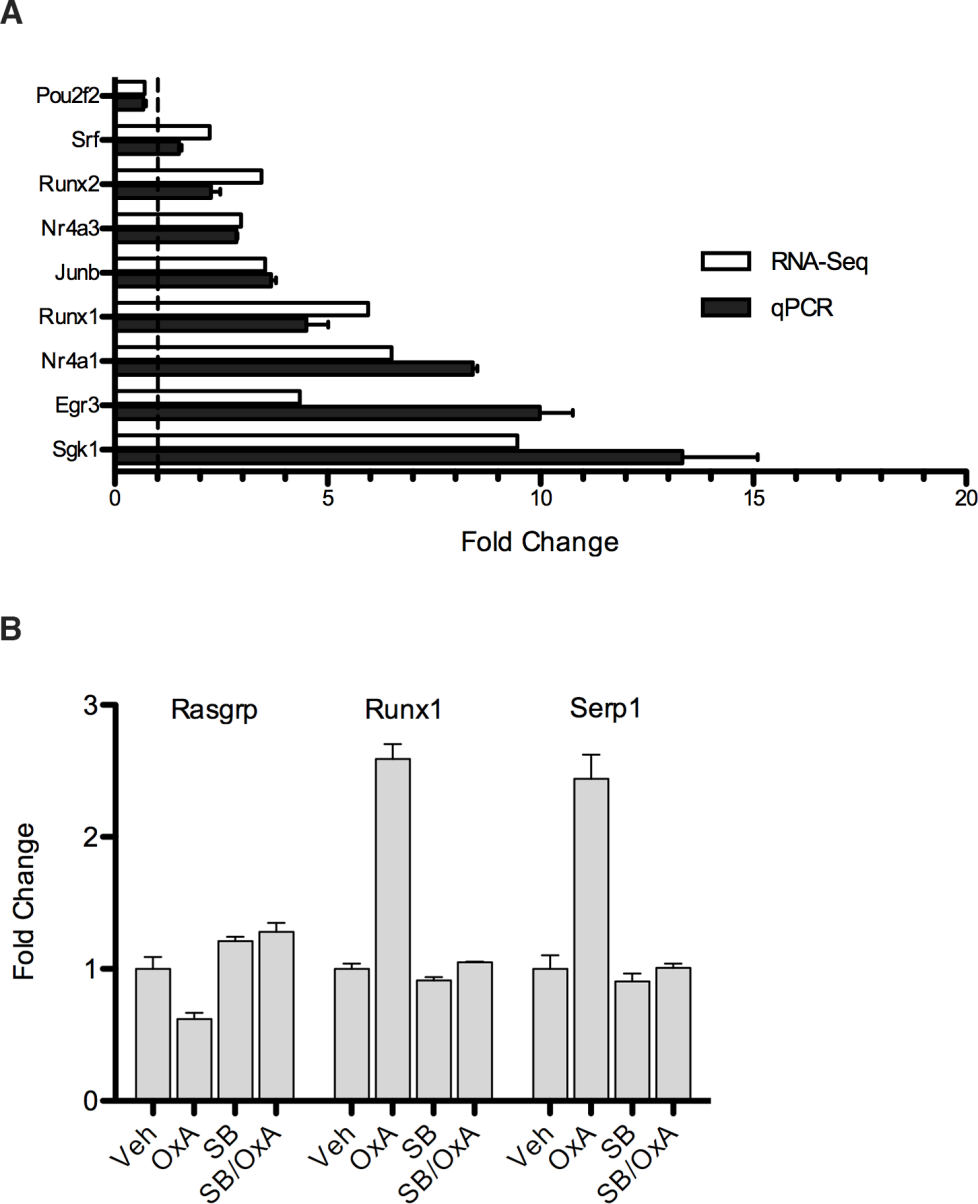
qPCR validation of RNA-Seq data. (A) A subset of genes that were differentially regulated by OX_1_ signaling in the RNA-Seq experiment were chosen for qPCR verification. GT1-7-OX_1_ cells were treated with 100nM OxA for 3 hours. The fold-change from the RNA-Seq data is included as a reference to demonstrate similarity of effects. (B) An OX_1_-specific antagonist inhibits OxA-dependent changes in transcription. GT1-7-OX_1_ cells were treated with 20μM SB-334867 for 10 minutes prior to addition of 100nM OxA for 3 hours. RNA was purified from each sample and analyzed via qPCR. Mean fold change is presented (n = 1, reads done in triplicate) with error bars representing SEM.

In order to validate the changes in gene expression demonstrated by the RNA-Seq data, the experiment was repeated and a subset of genes was analyzed by qPCR. The levels of gene expression measured were consistent with the RNA-Seq data. (Fig 4A). In a second, smaller validation experiment, SB-334867 was used to inhibit the orexin-dependent differential expression of another subset of genes, demonstrating that OX_1_ activation was required for these changes in transcription (Fig 4B).

The results were also compared to a microarray study of OX_1_-expressing HEK293 cells published previously [71]. Of the genes that were up-regulated 2-fold or greater by OX_1_ activation in HEK293 cells, 346 had gene symbols that were present in the GT1-7-OX_1_ RNA-Seq data. Of these, only 24 (6.9%) were similarly up-regulated in GTI-7-OX_1_ cells (2-fold or greater at 3 or 8h, adj. p <0.05). Of the genes that were down-regulated 2-fold or greater by OX_1_ activation in HEK293 cells, 370 had gene symbols that were present in the GT1-7-OX_1_ RNA-Seq data, with only 1 (0.3%) that was similarly down-regulated (2-fold or greater at 3 or 8h, adj. p <0.05). Conversely, 5 of the 370 (1.4%) down-regulated genes were actually up-regulated 2-fold or greater at either 3 or 8h in GT1-7-OX_1_ cells. So, while some similarities were observed, the data were poorly replicated in the two systems, highlighting the importance of using an appropriate model for studying signal transduction, *in vitro*. Nevertheless, we did identify a set of genes comparably regulated by OX_1_ in both HEK293 and GT1-7 cells, strongly supporting a role for these genes in OX_1_ signal transduction (S4 Table).

### Transcription factors regulated by OX_1_ signaling

GPCR-mediated signaling usually results in the activation or suppression of transcription factors, which then drive downstream changes in global gene expression. The data were thus searched for OX_1_-regulated genes known to encode transcription factors. Several families of related transcription factors, mostly immediate early genes, were highly regulated by orexin. These include the early growth response genes (*Egr1*, *Egr2*, *Egr3*), AP-1 (*Fos*, *Fosb*, *Junb*, *Fosl2*), high mobility group superfamily A (*Hmga1*, *Hmga2*), the Nr4a family of nuclear hormone receptors (*Nr4a1*, *Nr4a3*), the Id family of transcriptional repressors (*Id1*, *Id2*, *Id3*, *Id4*), various Kruppel-like factors and related proteins (*Klf4*, *Klf5*, *Klf6*, *Klf9*, *Klf10*, *Klf11*, *Klf13*, *Klf16*, *Glis1*, *Glis2*, *Zbtb16*), and the Runx family (*Runx1*, *Runx2*, *Runx1t1*) (Table 3).

**Table 3:** Partial list of transcription factors regulated by OxA in GT1-7-OX_1_ cells.

Gene ID	3h Log2 FC	3h Adj p-value	8h Log2 FC	8h Adj p-value
Egr1	1.63	4.15E-70	0.77	7.45E-15
Egr2	2.14	1.48E-42	1.13	1.32E-11
Egr3	2.12	6.03E-25	0.35	2.29E-01
Egr4	0.76	1.28E-03	0.37	NA
Fos	1.25	3.11E-14	0.69	1.19E-04
Fosb	3.01	1.26E-92	0.96	9.48E-09
Fosl2	1.50	5.66E-130	0.78	1.62E-34
Junb	1.82	3.99E-94	0.68	1.07E-12
Glis1	1.58	1.69E-17	0.05	8.95E-01
Glis2	0.60	5.96E-19	0.13	1.52E-01
Hmga1	1.75	5.81E-37	1.63	6.45E-32
Hmga2	2.11	2.28E-24	2.20	2.77E-26
Id1	1.01	3.29E-11	0.29	1.34E-01
Id2	0.68	1.36E-12	-0.28	1.28E-02
Id3	1.28	4.13E-33	0.11	5.08E-01
Id4	1.23	7.93E-11	0.30	2.38E-01
Klf4	1.06	2.96E-18	0.20	2.28E-01
Klf5	0.95	1.53E-07	0.47	2.48E-02
Klf6	1.00	2.01E-39	0.60	7.32E-14
Klf9	0.81	2.34E-44	-0.01	9.60E-01
Klf10	1.57	3.67E-70	0.53	5.29E-08
Klf11	-0.70	5.11E-17	-0.29	1.24E-03
Klf13	0.55	2.23E-21	0.17	1.13E-02
Klf16	0.95	8.55E-18	0.36	5.55E-03
Myc	2.21	1.60E-98	1.78	1.74E-62
Nr4a1	2.70	5.99E-71	1.49	2.45E-20
Nr4a2	-0.10	7.88E-01	-0.04	9.29E-01
Nr4a3	1.57	1.69E-12	0.26	4.28E-01
Runx1	2.57	6.96E-246	1.95	2.93E-138
Runx1t1	-1.13	1.17E-11	-0.62	3.23E-04
Runx2	1.79	3.17E-95	0.37	3.52E-04

In order to identify the transcription factors likely responsible for driving the more downstream changes in gene expression, we performed an *in silico* analysis with PSCAN, an automated program that examines the promoter regions of regulated genes in order to identify common transcription factor binding sites [72]. The PSCAN analysis identified several putative transcription factors but, when combined with the expression data, strongly suggested a role for two distinct families of transcription factors in the regulation of downstream genes, the early growth response (EGR) proteins and Kruppel-like factors (KLF), in addition to the Myc transcription factor (Table 4). Of note, the EGR genes are heavily associated with neural plasticity and memory [73] as well as enhanced long-term potentiation that impacts relapse in drug-related reward behaviors [74,75], processes in which orexin signaling is known to be involved [12,76–78].

**Table 4:** A partial set of transcription factors identified by PSCAN analysis of genes differentially regulated by OX_1_ signaling.

PSCAN Results from 3h OX_1_-Regulated Transcripts							PSCAN Results from 8h OX_1_-Regulated Transcripts						
TF_NAME	MATRIX_ID	Z_SCORE	P_VALUE	SAMPLE_SIZE	3h Log2 FC	8h log2 FC	TF_NAME	MATRIX_ID	Z_SCORE	P_VALUE	SAMPLE_SIZE	3h log2 FC	8h log2 FC
**EGR1**	MA0162.2	9.6026	2.84E-22	265	**1.63**	0.77	**EGR1**	MA0162.2	4.0123	2.94E-05	106	**1.63**	0.77
SP2	MA0516.1	8.6975	1.35E-18	265	0.18	-0.13	Myog	MA0500.1	3.9488	3.81E-05	106	0.00	0.07
SP1	MA0079.3	8.3484	2.91E-17	265	0.25	0.05	E2F3	MA0469.1	3.8741	5.20E-05	106	0.52	0.24
SP1	MA0079.2	8.2675	5.68E-17	265	0.25	0.05	Tcf12	MA0521.1	3.7984	7.08E-05	106	0.07	0.00
**EGR3**	MA0732.1	7.9222	8.97E-16	265	**2.12**	0.35	E2F4	MA0470.1	3.7032	1.04E-04	106	0.43	0.42
E2F3	MA0469.1	7.8067	2.35E-15	265	0.52	0.24	Ascl2	MA0816.1	3.6859	1.11E-04	106	0.02	-0.03
E2F1	MA0024.2	7.7749	2.98E-15	265	0.00	-0.04	E2F6	MA0471.1	3.5883	1.62E-04	106	0.34	0.19
**EGR2**	MA0472.1	7.6289	9.46E-15	265	**2.14**	**1.13**	E2F1	MA0024.2	3.4454	2.79E-04	106	0.00	-0.04
E2F4	MA0470.1	7.5845	1.34E-14	265	0.43	0.42	Tcf3	MA0522.1	3.3927	3.38E-04	106	-0.08	0.00
EGR4	MA0733.1	7.3161	1.04E-13	265	0.76	0.37	**EGR2**	MA0472.1	3.2724	5.23E-04	106	**2.14**	**1.13**
Tcfl5	MA0632.1	7.2269	2.01E-13	265	-0.01	0.06	Tcfl5	MA0632.1	3.2113	6.49E-04	106	-0.01	0.06
**Egr1**	MA0162.1	6.9829	1.17E-12	265	**1.63**	0.77	SP1	MA0079.2	3.2068	6.60E-04	106	0.25	0.05
KLF16	MA0741.1	6.8773	2.61E-12	265	0.95	0.36	MZF1	MA0056.1	3.1333	8.54E-04	106	-0.36	-0.13
TFAP2A	MA0003.1	6.8724	2.78E-12	265	0.04	0.16	KLF16	MA0741.1	3.1222	8.86E-04	106	0.95	0.36
GLIS2	MA0736.1	6.7071	8.43E-12	265	0.60	0.13	SP1	MA0079.3	3.1137	9.11E-04	106	0.25	0.05
KLF5	MA0599.1	6.6776	1.07E-11	265	0.95	0.47	SP3	MA0746.1	3.0765	1.03E-03	106	0.12	0.06
E2F6	MA0471.1	6.4745	3.96E-11	265	0.34	0.19	SP2	MA0516.1	2.9850	1.40E-03	106	0.18	-0.13
**EGR2**	MA0472.2	6.4703	4.10E-11	265	**2.14**	**1.13**	Mafb	MA0117.1	2.9324	1.65E-03	106	0.28	-0.24
TFAP2A	MA0810.1	6.4591	4.60E-11	265	0.04	0.16	GLIS2	MA0736.1	2.8684	2.03E-03	106	0.60	0.13
SP4	MA0685.1	6.4064	6.39E-11	265	-0.09	0.01	TBP	MA0108.2	2.8372	2.24E-03	106	0.08	0.12
SP3	MA0746.1	6.3527	9.30E-11	265	0.12	0.06	TBP	MA0108.1	2.7867	2.62E-03	106	0.08	0.12
HINFP	MA0131.2	6.3166	1.14E-10	265	0.09	-0.03	NHLH1	MA0048.2	2.7563	2.88E-03	106	-0.04	-0.04
TFAP2A	MA0003.3	5.8567	2.14E-09	265	0.04	0.16	SREBF2	MA0596.1	2.7123	3.29E-03	106	0.75	0.21
ZBTB33	MA0527.1	5.8357	2.32E-09	265	0.17	0.23	KLF5	MA0599.1	2.6803	3.64E-03	106	0.95	0.47
SP8	MA0747.1	5.8083	2.80E-09	265	0.02	-0.13	Arnt	MA0006.1	2.5469	5.35E-03	106	-0.23	-0.12
PLAG1	MA0163.1	5.7457	4.10E-09	265	-0.11	-0.06	**EGR3**	MA0732.1	2.5277	5.67E-03	106	**2.12**	0.35
**Hes1**	MA1099.1	5.6521	6.99E-09	265	**1.95**	0.34	MZF1	MA0057.1	2.4225	7.63E-03	106	-0.36	-0.13
TFAP2C	MA0814.1	5.5411	1.37E-08	265	0.09	-0.03	**EGR2**	MA0472.2	2.4220	7.63E-03	106	**2.14**	**1.13**
**Klf4**	MA0039.2	5.3886	3.23E-08	265	**1.06**	0.20	Gmeb1	MA0615.1	2.4089	7.90E-03	106	-0.04	-0.04
TFAP2A	MA0872.1	5.3834	3.26E-08	265	0.04	0.16	EGR4	MA0733.1	2.4006	8.09E-03	106	0.76	0.37
Mycn	MA0104.2	5.2427	6.93E-08	265	0.31	0.41	CTCF	MA0139.1	2.3520	9.23E-03	106	0.02	0.02
INSM1	MA0155.1	5.2409	7.15E-08	265	0.32	-0.37	NHLH1	MA0048.1	2.3479	9.34E-03	106	-0.04	-0.04
SP1	MA0079.1	5.2060	8.88E-08	265	0.25	0.05	MSC	MA0665.1	2.3283	9.81E-03	106	0.03	0.00
TFAP2C	MA0815.1	5.2006	8.92E-08	265	0.09	-0.03	**Hes1**	MA1099.1	2.3026	1.05E-02	106	**1.95**	0.34
CTCF	MA0139.1	5.1714	1.03E-07	265	0.02	0.02	NR2C2	MA0504.1	2.2793	1.12E-02	106	-0.03	-0.07
TFAP2C	MA0524.2	5.1544	1.14E-07	265	0.09	-0.03	INSM1	MA0155.1	2.2741	1.14E-02	106	0.32	-0.37
MZF1	MA0056.1	5.0663	1.90E-07	265	-0.36	-0.13	TFAP2A	MA0003.1	2.2472	1.22E-02	106	0.04	0.16
NFKB1	MA0105.2	4.7060	1.15E-06	265	0.28	0.15	NFKB1	MA0105.2	2.2462	1.22E-02	106	0.28	0.15
Gmeb1	MA0615.1	4.6534	1.47E-06	265	-0.04	-0.04	**Klf4**	MA0039.2	2.2182	1.32E-02	106	**1.06**	0.20
HEY1	MA0823.1	4.5599	2.35E-06	265	0.51	-0.19	SP8	MA0747.1	2.1886	1.42E-02	106	0.02	-0.13
ZBTB7A	MA0750.1	4.5509	2.47E-06	265	0.27	0.15	SREBF1	MA0595.1	2.1807	1.45E-02	106	-0.09	0.09
**Myc**	MA0147.1	4.4119	4.64E-06	265	**2.21**	**1.78**	PAX5	MA0014.2	2.1370	1.62E-02	106	0.00	0.03
Zfx	MA0146.2	4.3194	7.35E-06	265	0.22	0.05	Egr1	MA0162.1	2.0647	1.93E-02	106	**1.63**	0.77
NRF1	MA0506.1	4.3138	7.58E-06	265	0.06	0.05	ARNT	MA0259.1	2.0407	2.05E-02	106	-0.23	-0.12
Zfx	MA0146.1	4.3072	7.76E-06	265	0.22	0.05	TFAP2A	MA0003.3	2.0303	2.10E-02	106	0.04	0.16
ZBTB7B	MA0694.1	4.1942	1.27E-05	265	-0.10	-0.05	TFAP2C	MA0814.1	1.8857	2.95E-02	106	0.09	-0.03
ZBTB7C	MA0695.1	4.1463	1.58E-05	265	0.15	0.04	SP4	MA0685.1	1.8691	3.06E-02	106	-0.09	0.01
Pax5	MA0014.1	4.1160	1.78E-05	265	0.00	0.03	**BHLHE40**	MA0464.2	1.8338	3.31E-02	106	**1.27**	0.51
Arnt	MA0006.1	4.0922	1.97E-05	265	-0.23	-0.12	Pax2	MA0067.1	1.8106	3.48E-02	106	0.00	0.03
**GLIS1**	MA0735.1	4.0484	2.40E-05	265	**1.58**	0.05	USF1	MA0093.1	1.7638	3.86E-02	106	-0.37	-0.17

The PSCAN results implicate several transcription factors that could be driving OX_1_-dependent changes in gene expression, a number of which were highly regulated by OX_1_ activation. Taken together, the promoter analysis and the expression data strongly suggest roles for the Myc transcription factor, as well as the early growth response (EGR) and Kruppel-like factor (KLF) families, in OX_1_ signaling. Transcription factors that were differentially regulated approximately 2-fold or greater are highlighted in bold.

### Sleep deprivation-related genes

To assess the physiological relevance of the data presented above, we searched the literature to compare our results with those from animal studies focused on orexin-related behaviors. Interestingly, there are striking similarities between our data set and those from an SD computational meta-analysis aimed at identifying highly conserved SD-related genes [61]. In this study, the authors combined and analyzed data from all available SD microarray studies spanning four species (mouse, rat, sparrow, and fruit fly). They found that SD resulted in a highly conserved (across at least 3 species) induction of *Egr1*, *Nr4a1*, and *Arc*, all of which were induced strongly by OxA in GT1-7-OX_1_ cells. Furthermore, the authors reported a set of 91 mouse genes that were differentially regulated during short-term SD (zeitgeber time 0-6h). Of the 90 SD-related genes that could be cross-referenced to our data (see Materials and Methods), 45.6% (41/90) were differentially regulated in response to 3h OX_1_ signaling at statistically significant levels (adj. p-value <0.05, Table 5). Additionally, 33.3% of these genes (30/90) were regulated in the same fashion (up or down) in both studies. These genes notably included *Egr1*, *Egr2*, *Egr3*, *Nr4a1*, *Nr4a3*, *Arc*, and *Sgk1*. Since it is known that SD results in elevated levels of OxA in the locus coeruleus and hypothalamus [79], increased hypothalamic OxA immunoreactivity [80,81], and increased expression of c-fos in orexinergic neurons [82], these parallels between the data presented here and gene regulation in SD suggest strongly that the events occurring in OX_1_-expressing GT1-7 cells are of significant physiological relevance.

**Table 5:** Genes regulated by both sleep deprivation and OX_1_ signaling.

ProbeID	GeneSymbol	Wang log2 FC	3h Log2 FC	8h Log2 FC	3h adj-p	8h adj-p
1416041_at	SGK1	1.09	3.24	2.12	0.00E+00	1.76E-163
1418687_at	ARC	0.97	2.37	0.65	5.79E-71	1.25E-05
1437247_at	FOSL2	0.87	1.50	0.78	5.66E-130	1.62E-34
1424638_at	CDKN1A	0.79	0.95	0.64	3.00E-31	4.82E-14
1427683_at	EGR2	0.79	2.14	1.13	1.48E-42	1.32E-11
1425671_at	HOMER1	0.74	1.04	0.47	9.93E-41	2.57E-08
1416064_a_at	HSPA5	0.74	0.36	0.09	8.77E-15	1.52E-01
1416953_at	CTGF	0.70	0.47	0.21	4.35E-02	NA
1457472_at	GIGYF2	0.69	-0.21	0.05	3.16E-02	6.97E-01
1428112_at	MANF	0.67	0.22	0.08	3.19E-03	4.06E-01
1438796_at	NR4A3	0.66	1.57	0.26	1.69E-12	4.28E-01
1419874_x_at	ZBTB16	0.65	-1.01	-0.48	1.86E-18	4.21E-05
1428834_at	DUSP4	0.64	1.69	1.16	3.44E-14	7.95E-07
1417394_at	KLF4	0.64	1.06	0.20	2.96E-18	2.28E-01
1454725_at	TRA2A	0.62	0.28	0.13	1.44E-02	3.32E-01
1448352_at	LUZP1	0.61	0.45	0.22	1.25E-19	6.05E-05
1436329_at	EGR3	0.60	2.12	0.35	6.03E-25	2.29E-01
1416505_at	NR4A1	0.58	2.70	1.49	5.99E-71	2.45E-20
1417677_at	OPN3	0.58	0.44	0.31	3.04E-02	1.79E-01
1438201_at	PDP1	0.57	0.76	0.89	2.10E-15	6.32E-21
1423796_at	SFPQ	0.57	0.32	0.16	4.12E-06	4.88E-02
1417602_at	PER2	0.56	0.84	0.25	1.28E-09	1.64E-01
1439442_x_at	YARS2	0.55	0.32	0.35	4.93E-04	1.03E-04
1434595_at	TRIM9	0.52	1.00	0.18	5.76E-27	1.65E-01
1417065_at	EGR1	0.51	1.63	0.77	4.15E-70	7.45E-15
1438724_at	OSBPL3	0.51	0.21	0.43	4.56E-03	7.96E-11
1437868_at	FAM46A	0.50	0.39	0.33	4.49E-02	1.09E-01
1460672_at	2410002F23Rik	-0.51	0.42	0.35	5.36E-07	4.89E-05
1452661_at	TFRC	-0.51	0.63	0.43	1.31E-07	7.56E-04
1439503_at	ZFP28	-0.51	-0.34	-0.06	7.17E-03	7.34E-01
1422185_a_at	cyb5r3	-0.53	0.12	0.10	4.67E-02	1.55E-01
1426378_at	EIF4B	-0.53	0.30	0.27	4.74E-10	2.50E-08
1451566_at	Zfp810	-0.53	-0.61	-0.35	7.29E-05	3.68E-02
1421821_at	LDLR	-0.54	1.78	1.18	1.22E-186	6.26E-80
1421033_a_at	TCERG1	-0.54	0.38	0.19	1.03E-07	2.12E-02
1455017_a_at	ZMYM3	-0.54	-0.64	-0.49	5.61E-25	7.85E-15
1428630_x_at	HAGHL	-0.64	-0.38	-0.41	8.34E-03	4.65E-03
1429239_a_at	STARD4	-0.67	0.73	0.10	7.16E-12	5.61E-01
1449039_a_at	HNRNPDL	-0.73	0.28	0.15	3.06E-05	5.77E-02
1418174_at	DBP	-0.78	-0.86	-0.72	6.65E-06	1.98E-04
1434817_s_at	RPRD2	-0.78	0.14	0.09	3.72E-02	2.44E-01

A cross-comparison of genes identified in a sleep deprivation microarray meta-analysis with genes regulated by OX_1_ in GT1-7-OX_1_ cells indicated strong similarities between the data sets. The Gene Symbol and SD log_2_FC columns are from the Wang, et al. paper, while the other columns are from the current study. Values shaded in red are up-regulated while values shaded in green are down-regulated.

### Role of Sgk1 in orexin signaling

**Fig 5:**
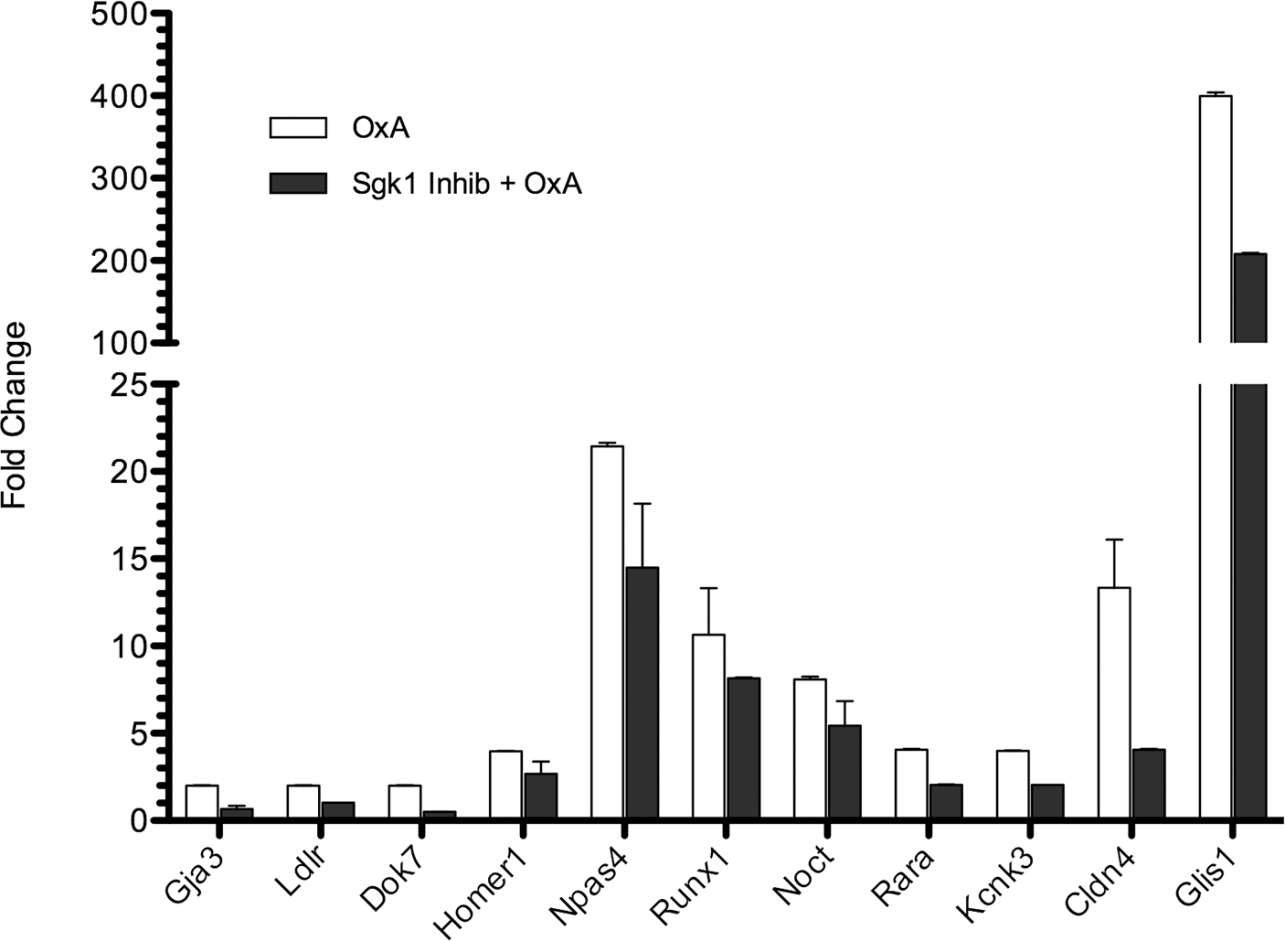
Inhibition of Sgk1 depresses the OxA-dependent induction of a small set of transcripts. GT1-7-OX_1_ cells were treated with an Sgk1 inhibitor, GSK-650394, prior to addition of OxA. Total RNA was purified from lysates and used for qPCR analysis. The genes displayed had their level of orexin-induced transcription inhibited by GSK-650394. Bars represent averages (n = 1, reads done in triplicate) while error bars represent SEM.

One of the most highly orexin-responsive genes in GT1-7-OX_1_ cells, serum/glucocorticoid-regulated kinase 1 (*Sgk1*), is also highly up-regulated by SD. This transcript was of particular interest due to its roles in neuronal excitation and synaptic plasticity [83–86]. Sgk1 is expressed in all tissues of the body, including the brain, and regulates numerous ion channels, molecular transporters, and signaling proteins [87–91]. Transcription of *Sgk1* mRNA has been shown to be induced by several stimuli and, as it relates to orexin, by exposure to drugs of abuse [92,93] or fasting conditions [94–96]. The *Sgk1* transcript was highly induced in response to OxA at both 3 and 8 hours (9-fold and 4-fold, respectively), with adj. p-values approaching zero. In order to examine the potential role of Sgk1 in orexin-regulated gene expression, an Sgk1 inhibitor, GSK-650394 [97], was added to GT1-7-OX_1_ cells prior to the addition of OxA. The effects of the Sgk1 inhibition on transcription of 89 of the most highly OX_1_-regulated genes were determined by qPCR (S2 Fig). From this set of genes, eleven showed reduced levels of induction when pretreated with GSK-650394 (Fig 5). These data argue that Sgk1 regulates these genes, possibly through phosphorylation of a transcription factor(s) that targets them. In an attempt to identify this putative factor(s), the PSCAN analysis was repeated with this set of genes (S5 Table). As seen in the earlier results, the Egr family of transcription factors was again identified. Interestingly, one of the other transcription factors identified, Sp1, is a known substrate of Sgk1 [98] and has been shown to regulate the transcription of *Ldlr* [99,100], *Dok7* [101], *Rara* [102,103], and *Cldn4* [104,105].

## Discussion

Because the orexin system has been shown to regulate behavior primarily via its actions in the central nervous system, studying orexin receptor signaling in a neuronal context is of particular interest. To that end, a recombinant model stably expressing OX_1_ was generated in GT1-7 cells, a mouse cell line derived from GnRH-expressing neurons of the hypothalamus. With this recombinant model established, RNA-Seq was used to identify a large set of genes regulated by OX_1_ signaling including several immediate early genes, transcription factors, kinases, and phosphatases. Results from qPCR validation experiments correlated well with the RNA-Seq data, affirming many of the transcriptional changes observed. In order to identify the primary transcriptional regulators or OX_1_ signaling, an *in silico* transcription factor binding site analysis was performed. While the occurrence of false positives is a legitimate concern when using these types of analyses, correlating these results with the gene expression data further supports roles for several of the transcription factors identified in the promoter analysis. Notably, these included related transcription factors such as *Egr1*, *Egr2*, and *Egr 3* as well as a number of Kruppel-like factors and the Myc transcription factor.

Of particular importance, though, is whether or not these data are physiologically relevant. To address this, the data were compared to those from similar studies focused on orexin-related behaviors, such as sleep and wakefulness. These comparisons demonstrated that OX_1_ signaling shows similarities with transcriptional profiles seen in *in vivo* SD microarray studies, with several genes being similarly regulated. This suggests that orexin signaling may be responsible for the SD-induced changes in expression of these genes. Indeed, SD has been shown to result in the activation of orexigenic neurons and increased OxA peptide levels in the brain, further supporting this hypothesis.

One of the genes highly regulated by both orexin signaling in GT1-7-OX_1_ cells and SD in the mouse brain is *Sgk1*, a kinase that is known to regulate a number of cellular proteins and is strongly associated with neuronal excitability, synaptic plasticity, and memory formation. As orexin also has strong associations with all of these processes, the role of Sgk1 in OX_1_ signaling seemed to be particularly interesting. These analogous roles imply that Sgk1 may be a crucial mediating factor in transducing the biological effects of orexin. A likely scenario is that, when induced by OX_1_ activation, Sgk1 phosphorylates cellular ion channels, altering the excitability of OX_1_-expressing neurons, thereby influencing associated behaviors.

In order to evaluate the downstream effects of Sgk1 on OX_1_ signaling, we analyzed the impact of Sgk1 inhibition on the orexin-dependent differential regulation of a subset of genes by qPCR. Inhibition of Sgk1 resulted in diminished induction of a small set of genes that included *Gja3*, *Ldlr*, *Dok7*, *Rara*, *Kcnk3*, and *Cldn4*, amongst others (Fig 5). Promoter analysis identified several putative transcription factors that could regulate these genes. Of these, Sp1 seems to be the most interesting, as it is known to be phosphorylated by Sgk1 and regulates transcription of many of the genes whose OX_1_-dependent transcription was impaired by Sgk1 inhibition, although the role of Sp1 was not empirically addressed in this study.

In summary, the orexin system sits at the crossroads of a diverse set of related behaviors including sleep, memory, synaptic plasticity, and addiction. To give insight into the molecular mechanisms influencing the role of orexin in regulating these behaviors, a neuronal, recombinant cell line was utilized to identify a set of candidate genes involved in OX_1_ signaling. Corroborating evidence in the literature strongly supports the physiological relevance of this data set, as several of the genes regulated by OX_1_ in the model cell line are similarly regulated by SD, *in vivo*. While this study brought focus to the role of Sgk1 in OX_1_ signaling, the data set is rich with other candidate genes whose capacity in OX_1_ signaling commands further study. Some examples include *Homer1*, *Arc*, *Nr4a1*, and *Ldlr*, each of which were heavily induced by OX_1_ signaling and have been associated with numerous orexin-related behaviors [73,93,106–110].

## Supporting information

S1 Fig Exploratory analysis. A principle component analysis plot (A) and hierarchal clustering dendrogram (B) each show clear separation between treatment groups and strong clustering of samples within a condition.
(TIF)

S2 Fig Effects of Sgk1 inhibition on OX_1_-regulated transcription. GT1-7-OX_1_ cells were treated OxA or with an Sgk1 inhibitor, GSK-650394, prior to the addition of OxA. A set of 89 OX_1_-regulated transcripts was assayed via qPCR. Data were analyzed by the 2^-ΔΔC^_T_ method using *B2m* as the endogenous control and are represented as fold-change over control samples (n = 1, reads done in triplicate).
(TIF)

S1 Table List of TaqMan probes used in this work. For each probe, the Applied Biosystems catalog number is given.
(XLSX)

S2 Table Gene ID conversions. The complete list of genes that were regulated 2-fold or greater, at 3h or 8h, by OxA in GT1-7-OX_1_ cells were entered into the DAVID Gene ID Conversion Tool to generate RefSeq mRNA IDs (See Methods). The RefSeq mRNA ID’s were then entered into PSCAN for promoter analysis.
(XLSX)

S3 Table List of primer pairs used for the Sgk1 inhibition qPCRs. The complete set of 96 primer pairs includes 89 of the most highly OX_1_-regulated genes plus 7 housekeeping genes (*Actb*, *B2m*, *Gusb*, *Polr2a*, *Ppia*, *Rplp0*, and *Tbp*).
(CSV)

S4 Table OX_1_-regulated genes. A list of genes that were differentially regulated by OX_1_ signaling in both HEK293 and GT1-7 cells. The HEK293 Fold Up and Fold Down columns are from the Sikder, et. al paper while the remaining data are from this study.
(XLSX)

S5 Table Partial PSCAN results for the putative Sgk1-regulated genes. A PSCAN promoter analysis was performed using the eleven genes whose orexin-dependent induction was reduced by pre-incubation with the Sgk1 inhibitor.
(XLSX)
