# Supplementary figures and images for: Global analysis of gene expression mediated by OX1 orexin receptor signaling in a hypothalamic cell line
Source: PLoS One. 2017 Nov 16;12(11):e0188082. doi: 10.1371/journal.pone.0188082 (PMC5690679; doi:10.1371/journal.pone.0188082)

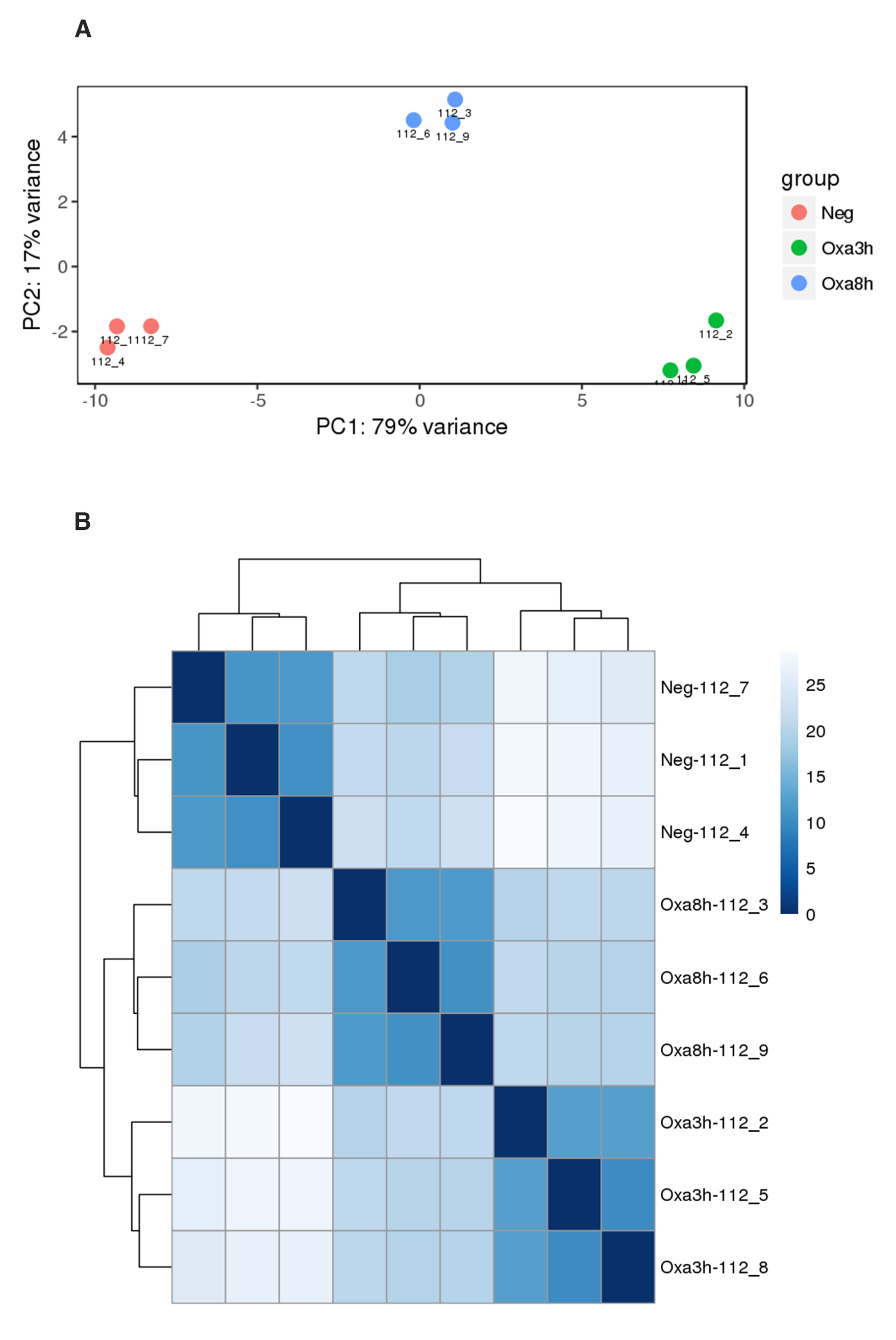

Supplement: S1 Fig — A principle component analysis plot (A) and hierarchal clustering dendrogram (B) each show clear separation between treatment groups and strong clustering of samples within a condition. (TIF) [file pone.0188082.s001.tif]

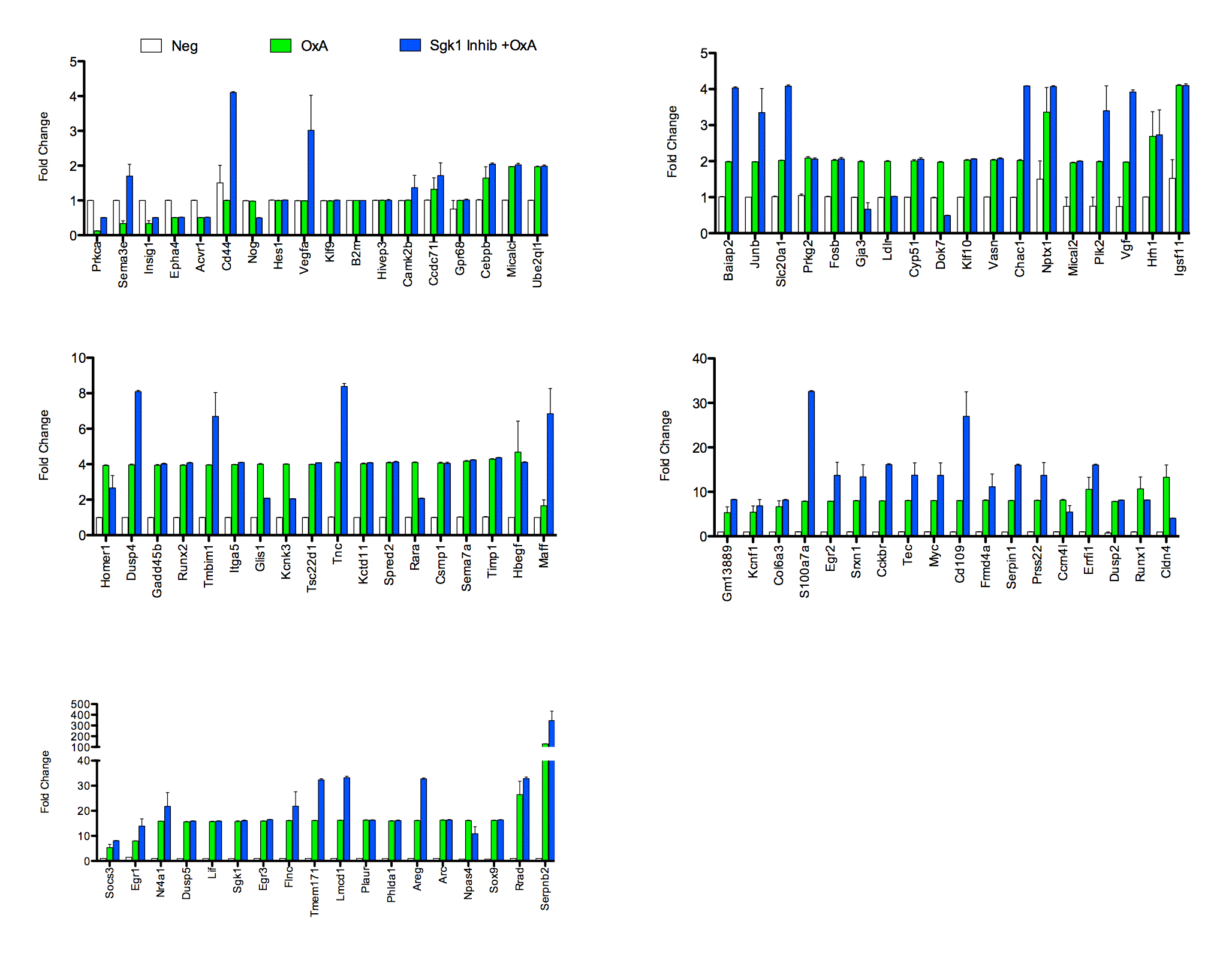

Supplement: S2 Fig — GT1-7-OX1 cells were treated OxA or with an Sgk1 inhibitor, GSK-650394, prior to the addition of OxA. A set of 89 OX1-regulated transcripts was assayed via qPCR. Data were analyzed by the 2-ΔΔCT method using B2m as the endogenous control and are represented as fold-change over control samples (n = 1, reads done in triplicate). (TIF) [file pone.0188082.s002.tif]
